# Human umbilical cord mesenchymal stem cells reverse depression in rats induced by chronic unpredictable mild stress combined with lipopolysaccharide

**DOI:** 10.1111/cns.14644

**Published:** 2024-03-03

**Authors:** Pengxiang Wang, Yunxia Li, Yongli Song, Yuan Gao, Chunxia Hao, Yang Zhou, Siqin Bao, Jitong Guo, Xihe Li

**Affiliations:** ^1^ The State Key Laboratory of Reproductive Regulation and Breeding of Grassland Livestock Inner Mongolia University Hohhot China; ^2^ Research Center for Animal Genetic Resources of Mongolia Plateau College of Life Sciences, Inner Mongolia University Hohhot China; ^3^ College of Basic Medicine, Inner Mongolia Medical University Hohhot China; ^4^ Inner Mongolia Saikexing Institute of Breeding and Reproductive Biotechnology in Domestic Animal Hohhot China; ^5^ Inner Mongolia Yihong Medical Research Co. Ltd Hohhot China

**Keywords:** human umbilical cord mesenchymal stromal cells, inflammation, metabolomics, proteomics, rat depression model, transcriptomics

## Abstract

**Background:**

Inflammation and oxidative stress are considered crucial to the pathogenesis of depression. Rat models of depression can be created by combined treatments of chronic unpredictable mild stress (CUMS) and lipopolysaccharide (LPS). Behaviors associated with depression could be improved by treatment with mesenchymal stem cells (MSCs) owing to immunomodulatory functions of the cells. Therapeutic potentials of the MSCs to reverse pro‐inflammatory cytokines, proteins, and metabolites were identified by transcriptomic, proteomic, and metabolomic analysis, respectively.

**Methods:**

A depression model was established in male SD rats by 2 weeks of CUMS combined with LPS. The models were verified by behavioral tests, namely SPT, OFT, EPM, and qRT‐PCR for pro‐inflammatory cytokines. Such depressed rats were administered human umbilical cord MSCs (hUC‐MSCs) via the tail vein once a week for 2 and 4 weeks. The homing capacity was confirmed by detection of the fluorescent dye on day 7 after the hUC‐MSCs were labeled with CM‐Dil and administered. The expression of GFAP in astrocytes serves as a biomarker of CNS disorders and IBA1 in microglia serves as a marker of microglia activation were detected by immunohistochemistry at 2 and 4 weeks after final administration of hUC‐MSCs. At the same time, transcriptomics of rat hippocampal tissue, proteomic and metabolomic analysis of the serum from the normal, depressed, and treated rats were also compared.

**Results:**

Reliable models of rat depression were successfully induced by treatments of CUMS combined with LPS. Rat depression behaviors, pro‐inflammatory cytokines, and morphological disorders of the hippocampus associated with depression were reversed in 4 weeks by hUC‐MSC treatment. hUC‐MSCs could reach the hippocampus CA1 region through the blood circulation on day 7 after administration owing to the disruption of blood brain barrier (BBB) by microglial activation from depression. Differentiations of whole‐genome expression, protein, and metabolite profiles between the normal and depression‐modeled rats, which were analyzed by transcriptomic, proteomics, and metabolomics, further verified the high association with depression behaviors.

**Conclusions:**

Rat depression can be reversed or recovered by treatment with hUC‐MSCs.

## Graphical abstract

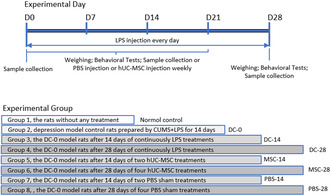

## 1 INTRODUCTION

Major depressive disorder (MDD) is a common debilitating psychiatric disorder that is characterized by depressed mood, diminished interests, sleep disturbances, poor concentration, impaired cognitive function, and suicidal tendencies.^1^ MDD is now a significant contributor to the social burden of disease, which affects about 5% of the worldwide population according to the World Health Organization's reports. The incidence of MDD has been estimated to increase by 27.6% owing to the COVID‐19 pandemic.^2^ Some pessimistic scenarios estimate that by the end of 2030, MDD would become the leading cause of disease burden globally. Stress was not traditionally recognized as an immune system activator. Since the first evidence reported that MDD is accompanied by activated immune‐inflammatory pathways with increased levels of pro‐inflammatory cytokines in 1990,^3^ increasing evidence has now indicated that stress exposure stimulates release of cytokines leading to inflammation.^4^ Importantly, the interaction between inflammation and depression has also revealed that an elevated level of cytokines can produce depressive symptomatology.^5^, ^6^ Although there are plenty of antidepressants currently available, most pharmacologic interventions take weeks to months to be effective and have low response rates.^7^

Currently, the chronic unpredictable mild stress (CUMS) model is widely used to investigate the mechanism and etiology of the animal model of depression,^8^ because CUMS can lead to immunoinflammatory activation, which is known to induce psychiatric disorders.^6^ However, the quality of a CUMS model can only be elevated by trained laboratory operators. Based on the high‐density stimulation, the mortality of model animals is very high and different experimental environments may lead to errors in results.^9^ Lipopolysaccharide (LPS) produced by most gram‐negative bacteria is commonly referred to as endotoxins known to stimulate glial cells, mainly microglia, to express several pro‐inflammatory genes to induce MDD.^10^ Thus, an MDD model can be induced by repeated and intermittent administration of LPS.^11^ An LPS‐induced depression‐like mouse model is also employed to investigate the mechanisms of inflammation‐associated depression and the therapeutic effects of drugs. Nevertheless, LPS‐induced depression‐like models varied widely in the characteristics and methodological parameters. In addition, LPS can induce other neurodegenerative disorders such as Alzheimer's disease (AD),^12^ Parkinson's disease (PD),^13^ Huntington's disease (HD),^14^ and amyotrophic lateral sclerosis (ALS)^15^ except for the depression model. Based on these studies, a reliable method to specifically create a depression model with CUMS and LPS needs further investigation.

Microglia reside in the central nervous system (CNS) and have been considered to originate from primitive macrophages^16^ that are crucially involved in CNS development, maturation, and aging.^17^, ^18^, ^19^ Moreover, microglia are highly associated with a rapid and robust response to disease and injury. Microglia interact with neurons to maintain neuronal functions in normal conditions and promote tissue repair after a CNS injury. Microglia downregulate the gene expression derived from CNS injury and destruction and change their morphology to promote tissue repair.^20^, ^21^ Conversely, some studies have suggested that activated microglia promote neuroinflammation occurrence and aggravate neuronal injury.^22^ Microglia activation can release pro‐inflammatory cytokines to affect tissue integrity, but these cells can also deviate from this phenotype to contribute to tissue repair and regeneration.^20^ These effects of microglia implied that the dual phenotype could skew pro‐inflammatory microglia toward an anti‐inflammatory phenotype which would have implications for CNS disease therapies. The molecular mechanisms of the phenotypic changes induced by microglial activation in neurological pathogenesis and protection are yet unclear. Indeed, microglial activation has also been clinically observed in patients with depression and might be interpreted as the reason for suicide.^23^ Microglial activation in depression models induced by stressful stimuli through a complex network of pathways has resulted in the release of immune molecules, including cytokines, chemokines, and reactive oxygen species.^24^ Whether pathogenesis and recovery of depression are mediated by pro‐inflammatory and anti‐inflammatory cytokines secreted by microglia remains to be further investigated.

Mesenchymal stem cells (MSCs) have been used in a significantly high number of clinical trials since its first therapeutic use in 1995.^25^, ^26^ The human umbilical cord MSCs (hUC‐MSCs) are a promising candidate for cell‐based therapy owing to its excellent advantages in regulating innate and adaptive immune systems to improve inflammation.^27^, ^28^ The immunomodulatory and anti‐inflammatory capabilities of hUC‐MSCs have also been recognized to improve neurological disorders, including LPS‐induced neuroinflammation and brain injury,^29^ CUMS‐induced depression, and stress‐related neurodegenerative disorders.^30^, ^31^ Numerous studies have revealed that the anti‐inflammatory action of hUC‐MSCs is accomplished through inhibiting the expression of inflammatory factors.^32^

In the present study, treatments of CUMS combined with LPS have been employed to create reliable and effective rat depression models. Subsequently, we evaluated the therapeutic potential of hUC‐MSCs by comparing the hippocampal whole‐genome transcriptome and hippocampal microglia morphological changes in the rat depression model bofore and after hUC‐MSCs treatment, and the correlation between depression and infammatory cytokines (IL‐6, TNF‐α, IL‐1β) in rats was investigated as well. In addition, proteomics and metabolomics analysis from the serum of depression rats were performed before and after hUC‐MSC treatments to confirm the differentially expressed proteins and metabolites, respectively, associated with depression behaviors in rat models. Accordingly, the molecular mechanisms and marker genes involved in the pathogenesis of rat depression models were investigated and scanned to provide evidence for further validation of stem cell treatment and diagnosis of depression.

## 2 METHODS

### 2.1 Animals

All animal experiments were approved and performed according to the guidelines of the Ethics Committee of Inner Mongolia University, Hohhot, China. Six‐week‐old, male Sprague‐Dawley (SD) rats (weight: 160–180 g) were purchased from Beijing Vital River Laboratory Animal Technology Co., Ltd. (Beijing, China) and housed under a 12‐h light/dark cycle in cages at 22°C, with ad libitum access to food and water. Rats were allowed to adapt to the new housing conditions for 1 week prior to commencement of experiments.

### 2.2 Creation of rat depression models

Rat depression‐like behaviors were induced with intraperitoneal injection of 0.5 mg/kg LPS (L5418, Sigma‐Aldrich, USA) at 9:00 a.m. daily for 2 weeks based on previously reported studies,^33^, ^34^ and CUMS treatments were modified from previous reports^35^ and performed during the time of LPS injections (Figure S2a) and finished within 2 h. The rats were chronically exposed to various randomly scheduled, low‐intensity, social, and environmental stressors for CUMS treatments. The stressors comprised 10 stimuli, namely isolation (as a continuous social stressor), tightness of the tail (for 1 min), deprivation of food or water (for 24 h), foreign matter in cage (for 24 h), heat stimulation (45°C water for 5 min), cage‐tilting (45° for 24 h), wet bedding (200 mL water per cage for 24 h), removal of sawdust bedding (for 24 h), continuous light (for 24 h), and horizontal shaking (for 5 min). Out of the nine stimuli, one was randomly or orderly selected for CUMS treatment per day within the 2 weeks. The same stressor was not applied for 3 consecutive days, to ensure that the rats could not predict the occurrence of stimulation.^36^

### 2.3 Behavioral measurements

All behavioral experiments except for sucrose preference test (SPT) were performed in a blinded fashion. Rats were allowed to adapt to the environment for 5 min. A video camera was placed above the test field, and the rat movement was captured for 5 min. Body weight (BW) for rats in each group were measured every week, and behavioral experiments were performed before sacrifice between 08:00 and 12:00 a.m.

The sucrose preference test (SPT) was used for evaluation of potential anhedonia in the experimental rats. SPT was modified and performed according to the published protocol.^37^, ^38^ Briefly, rats were given one bottle of 1% sucrose solution (w/v) and one bottle of tap water with ad libitum access to food during adaptation for the first 72 h. Afterward, one of the bottles was replaced with water for 24 h. The bottles were switched after 12 h during this 24 h period. Following this procedure, the rats were deprived of water and food for 24 h and then kept in individual cages with access to one bottle of 200 mL 1% sucrose solution and one bottle of 200 mL tap water. The SPT was conducted by measuring sucrose and water consumption after 24 h.

The open‐field test (OFT) was performed to assess the spontaneous exploratory activity of the rats.^38^ The open‐field arena was a square measuring 625 × 740 × 510 mm (L × W × H) with a high black floor divided into nine equally sized squares. A digital camera was placed 2 m above the open field to capture the whole field. During the 5 min observation period of OFT, the rats were placed at one center facing the wall, and the total moved distance and duration were recorded. The behaviors of the rats were analyzed by the animal behavior analysis system (Chengdu Taimeng Software Co., Ltd., China).

The elevated plus maze (EPM) was used to measure anxiety‐related behaviors in rodents.^39^ The apparatus was composed of two open arms (425 × 145 mm) and two opposite closed arms surrounded by 225 mm high walls of the same dimensions. The maze was elevated 500 mm above the ground, and the open arms were equipped with 5 × 5 mm ledges to ensure that no animals would fall off the maze. Rats were placed at the center facing an open arm and allowed to explore for 5 min. The time spent and distance traveled in the closed and open arms were recorded and calculated.

### 2.4 Culture and characterization of hUC‐MSCs

Isolation, culture, and characterization of hUC‐MSCs were modified from the published protocols.^40^ Donated human umbilical cords were collected from the placenta side after delivery in accordance with local laws and guidelines, which has been approved by the ethics committee of the First Hospital of Inner Mongolia Medical University. The cord was cut into 0.5 cm^3^ pieces and cultured in DMEM/F12 (Gibco) with 10% fetal bovine serum (Gibco) and 1% penicillin/streptomycin (Gibco) at 37°C in a humidified environment containing 5% CO_2_ in cell culture flasks to allow for cell migration and proliferation. The explant outgrowths were dissociated with TrypLE Select (1X) (Gibco) when they were almost confluent, and the cells were harvested by centrifugation at 1000 *g* for 5 min. The resuspended cells were passaged and cultured in DMEM/F12 for further experiments.

Characterization of hUC‐MSCs was performed by flow cytometry analysis of CD34, CD14, CD19, CD45, CD73, CD90, CD105, and HLA‐DR (BioLegend, California, USA), which were considered as MSC‐related surface markers, osteogenesis, chondrogenesis and adipogenesis potentials of hUC‐MSCs were evaluated after differentiation culture. Briefly, the hUC‐MSCs were dissociated into single cells by TrypLE Select (1X), resuspended in phosphate‐buffered saline (PBS, Gibco) at passage 3 (P3), and stained with the indicated antibodies against the surface markers. After washing with phosphate‐buffered saline (PBS), the cells were analyzed by FACS Canto II (BD Biosciences, USA) following previously described protocols. When the passaged cells reached 80% confluence, the culture medium was replaced with osteogenic (MSCgo™ Rapid Osteogenic Differentiation Medium, BI), chondrogenic (MSCgo™ Chondrogenic Differentiation Medium, BI), and adipogenic (MSCgo™ Adipogenic Differentiation Medium, BI) differentiation media, respectively. The medium was changed every 3 days following previously described protocols.^41^ The hUC‐MSC‐differentiated cells were stained by Alizarin Red, Alcian Blue, and Oil Red O staining and photographed under the microscope (Fluo‐View‐FV1000, Olympus, Japan). Karyotyping of the hUC‐MSCs was also performed at P3 to monitor the genomic stability. Metaphases of the hUC‐MSCs were arrested by incubation with Colcemid solution (KaryoMAX, Gibco) for 2 h at 37°C, and the cells were stained with Giemsa (KaryoMAX, Gibco) for karyotyping following the Giemsa banding procedure.^42^ The metaphase was captured by a microscope, and the karyotype was analyzed. Additionally, the Novogene software (https://magic.novogene.com/) was used to assess the statistical enrichment of differentially expressed genes or target genes in the Kyoto Encyclopedia of Genes and Genomes (KEGG) pathways (https://magic.novogene.com/customer/ngs‐kegg/).

### 2.5 Administration of hUC‐MSCs

All rat depression models were successfully induced by treatment of CUMS combined with LPS and were continuously stimulated with administration of 0.5 mg/kg LPS daily afterward. The hUC‐MSC treatments were performed at P3, and the cells were dissociated by TrypLE Select and resuspended in PBS at a density of 1.0 × 10^7^/mL. The rats were administered 0.5 mL PBS containing 5 × 10^6^ cells, each via the tail vein for hUC‐MSC treatment. The controls only received 0.5 mL PBS sham injection. The rats used for this experiment were randomly grouped into eight groups of six rats each: Group 1, NC group, in which the rats have did not receive any treatment; Group 2, depression control group at day 0, in which the rats were euthanized after 14 days of treatments with CUMS combined with LPS for creation of the depression models and were marked as group DC‐0; Group 3, depression control group at day 14, in which the rats were killed after they were continuously injected with LPS for 14 days after depression models were successfully created and marked as group DC‐14; Group 4, depression control group at day 28, in which the rats were killed after they were continuously injected with LPS for 28 days after depression models were successfully created and marked as group DC‐28; Group 5, hUC‐MSC treatment group at day 14, in which the depression model rats were administered with hUC‐MSCs once a week for 2 weeks and the rats were killed at 7 days after treatment and marked as group MSC‐14; Group 6, hUC‐MSC treatment group at day 28, in which the depression model rats were administered with hUC‐MSCs once a week for 3 weeks and then killed at 7 days after the last treatment and marked as group MSC‐28; Group 7, PBS sham injection control group at day 14, in which the depression model rats were administered with PBS once a week for 2 weeks and the rats were killed at 7 days after the last treatment and marked as group PBS‐14; and Group 8, PBS sham injection control group at day 28, in which the depression model rats were administered with PBS once a week for 3 weeks and the rats were killed at 7 days after the last treatment and marked as group PBS‐28.

### 2.6 Serum and tissue collection

The rats were anesthetized with tribromoethanol (Nanjing Aibei Biotechnology Co., Ltd) after 12 h of fasting and then killed. Blood sample was collected through cardiac puncture following a previously described protocol,^43^ and the serum was harvested by centrifugation at 1500 *g* at 4°C for 15 min. The serum samples were stored at −80°C until further analysis. The hippocampus was identified and collected by dissection from removing the cerebral cortex of each hemisphere.^44^ Half of the hippocampus was fixed in 4% paraformaldehyde (Fixative Solution, Gibco) for sectioning. The other half was immediately transferred into a pre‐chilled frozen vial and snap‐frozen in liquid nitrogen and then stored at −80°C for RNA profiling.

### 2.7 Histotomy and immunohistochemistry of the hippocampus

Hippocampal tissues were dehydrated and embedded in paraffin after fixation as described previously for hematoxylin‐eosin (HE) staining and immunohistochemistry.^45^ The paraffin‐embedded tissues were sectioned at a thickness of 5 μm. Some of the sections were stained with HE according to the standard protocol, and the morphology and structure of neurons in the CA1 region of the hippocampus were observed under a light microscope. Some sections were incubated with rabbit mAb anti‐GFAP (#80788, Cell Signaling Technology) and anti‐IBA1 (ionized calcium binding adaptor molecule 1; #17198, Cell Signaling Technology) and then incubated with Alexa Fluor 488 (#4412, Cell Signaling Technology) and 594 (#8889, Cell Signaling Technology) conjugated with goat anti‐rabbit IgG at room temperature for 2 h. The photomicrographs of the sections were captured by laser scanning confocal microscopy (Fluo‐View‐FV1000, Olympus, Japan), astrocytes and microglia morphologies were categorized with ImageJ protocols.^46^

### 2.8 Quantitative real‐time polymerase chain reaction (qRT‐PCR)

Total RNA was extracted from frozen hippocampal tissue using Eastep® Super Total RNA Extraction Kit (Promega, China) according to the manufacturer's instructions. cDNA synthesis and qRT‐PCR were performed with HiScript Q RT SuperMix for qPCR (Vazyme, China) according to the kit instructions. Relative mRNA expression levels were determined with KAPA SYBR® FAST qPCR Kits (KK4601, Roche) by using a thermal cycler (Thermo Fisher Scientific Inc.). All samples were run in triplicate, and the glyceraldehyde phosphate dehydrogenase (*GAPDH*) gene served as the internal reference.

### 2.9 Transcriptome

The transcriptome of the hippocampus derived from the rat model of depression was analyzed before and after hUC‐MSC treatment.^47^ Briefly, the total RNA from the hippocampus tissue was extracted with the same qRT‐PCR method described above, and mRNA was purified from total RNA using oligo(dT)‐magnetic beads (Dynabeads™ Oligo(dT)25, Life Technologies) according to the manufacturer's instructions. Sequencing libraries were generated by using NEBNext® Ultra™ RNA Library Prep Kit for Illumina® (NEB, USA) according to the manufacturer's protocol. Briefly, the mRNA was fragmented by divalent cations under elevated temperature in First Strand Synthesis Reaction Buffer (5×). First‐strand cDNA was synthesized using random hexamer primer and M‐MuLV Reverse Transcriptase (RNase H), and the second‐strand cDNA was subsequently synthesized using DNA Polymerase I and RNase H. Remaining overhangs were converted into blunt ends through exonuclease/polymerase activities. The 3′ ends of DNA fragments were adenylated and ligated to NEBNext Adaptor with hairpin loop structure to prepare for the hybridization step. The library fragments were purified with AMPure XP system (Beckman Coulter, Beverly, USA) for selection of cDNA fragments preferentially measuring 370–420 bp. PCR was performed with Index (X) Primer, Universal PCR primers, and Phusion High‐Fidelity DNA polymerase. Finally, PCR products were purified by AMPure XP system, and the library quality was assessed using the Agilent Bioanalyzer 2100 system. The cluster and sequence of samples was performed using TruSeq PE Cluster Kit v3‐cBot‐HS (Illumina) on a cBot Cluster Generation System by Novogene (Beijing, China) according to the manufacturer's instructions. Finally, the 125 bp/150 bp paired‐end reads were generated and sequenced on an Illumina HiSeq platform. Gene Ontology (GO) R package, an international standardized gene functional classification system for describing the properties of genes and their products in any organism, was used for the analysis of differentially expressed genes (DEGs).^48^

### 2.10 Metabolome

The metabolome of the rat model of depression was performed before and after hUC‐MSC treatment as reported previously.^49^ Briefly, the serum samples were mixed with methanol extraction including isotopically labeled internal standard mixture. The serum supernatant was collected after vortexing, sonication, and centrifugation. Proteomics analysis was carried out by using liquid chromatography with tandem mass spectrometry (LC‐MS/MS) on the Xevo G2‐XS QToF system (Waters, USA). Quality control (QC) samples were prepared by mixing equal volumes of extraction solution for all samples. The supernatant from each serum sample was analyzed separately using both positive and negative ion electrospray ionization (ESI) mode. Metabolite identification was performed by searching the Human Metabolome Database (HMDB) using the Progenesis QI metabolomics data processing software (https://hmdb.ca/metabolites/).^50^ The orthogonal partial least squares discriminant analysis (OPLS‐DA) was used to identify the sample distribution. The significantly different metabolites were selected based on the variable importance in the project (VIP) obtained by the OPLS‐DA and p‐value of Student's *t*‐test. The metabolites with VIP > 1 and *p* < 0.05 after correction were considered to indicate statistical significance.

### 2.11 Proteome

The proteome of the rat model of depression was analyzed before and after hUC‐MSC treatment.^51^ Briefly, high‐abundance proteins and proteins larger than 30 kDa in the serum were depleted by using Nanosep™ centrifugal devices with Omega™ membrane 30 K (OD030C34, Pall). The proteins larger than 10 KDa and smaller than 10 KDa were separated and collected using Nanosep™ centrifugal devices with Omega™ membrane 10 K (OD010C34, Pall), respectively. The protein samples were then subjected to LC‐MS/MS analysis using Orbitrap Exploris™ 240 Mass Spectrometer (Thermo Scientific). Quantitative proteomics was acquired by Data Dependent Acquisition (DDA) mode.

### 2.12 Statistical analysis

Statistical differences were determined by IBM SPSS software (version 22, IBM Inc.). All values are expressed as the mean ± standard error of mean (SEM). Body weight and sucrose preference were assessed using two‐way repeated‐measures analysis of variance (ANOVA) with one between‐subjects factor (group) and one within‐subjects factor of time (day). Other data were analyzed using one‐way ANOVA and Tukey's post‐hoc test for multiple comparisons of means. The Shapiro‐Wilk test was employed to assess the normality of the experimental data. Differences with a value of *p* < 0.05 were considered statistically significant.

### 2.13 Ethics

All animal experiments were performed using humane methods in compliance with the animal ethical standards approved by the Animal Research Committee of Inner Mongolia University, China.

## 3 RESULTS

### 3.1 Characterization of hUC‐MSCs

**FIGURE 1:**
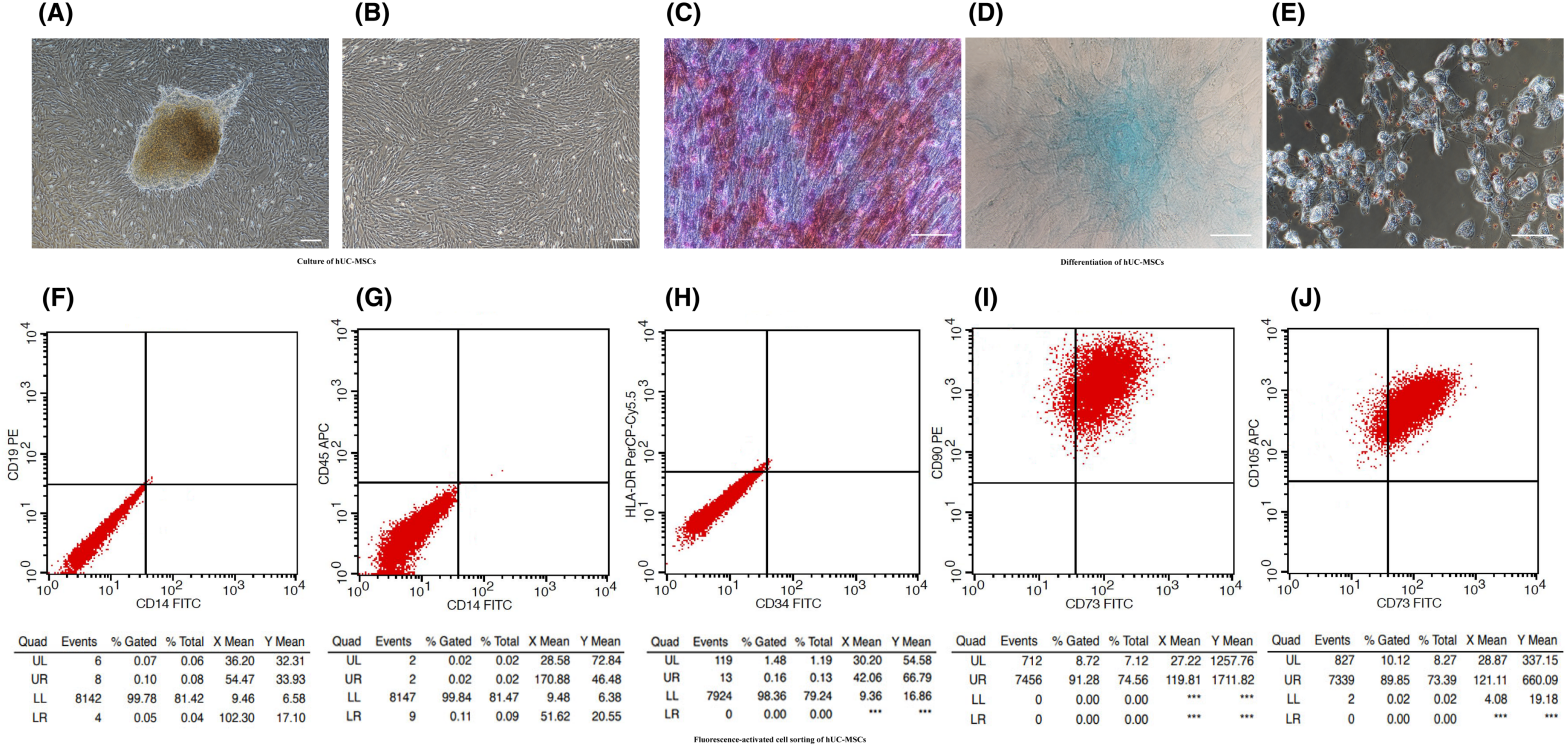
Characterization of hUC‐MSCs. The formation of heterogeneous populations of hUC‐MSCs with fibroblastic morphologies at passages P0 (A) and P3 (B). Scale bar: 100 μm. Bright‐field images for cell morphology and staining of osteogenic derivatives with calcium deposition, which was stained orange‐red by Alizarin Red (C), chondrogenic derivatives with formation of proteoglycans, which was stained blue by Alcian Blue (D), and adipogenic derivatives with formation of lipid droplets, which was stained red in Oil Red O (E). Scale bar: 200 μm. Flow cytometric analysis of the expression of cell surface markers, CD14 (0.15%), CD19 (0.17%), CD34 (0.16%), CD45 (0.04%), and HLA‐DR (1.64%) were negative, and CD73 (91.28%), CD105 (99.97%), and CD90 (100%) were positive (F‐J). Unstained cells for each condition were used as the negative controls.

The MSC outgrowths derived from explant culture of the human umbilical cord and passaged cells exhibited fibroblast‐like morphologies (Figure 1A,B) and S‐shaped cell growth curve (Figure S1a); no mycoplasma contamination was observed (Figure S1b), and normal chromosome karyotypes were observed at P3 (Figure S1c‐f). Histochemical staining showed that such hUC‐MSCs could differentiate into osteocytes after conditioned culture for 14 days in osteogenic media, while differentiate into chondrocyte and adipocytes after conditioned culture for 21 days in adipogenic and chondrogenic media. Osteogenic, chondrogenic, and adipogenic differentiation resulted in the deposition of calcium minerals, formation of proteoglycans and lipid vacuoles that were stained red with Alizarin Red, blue with Alcian Blue, and red with Oil Red O, respectively (Figure 1C–E). Flow cytometry results showed that the hUC‐CMSCs were highly negative for CD14 (0.15%), CD19 (0.17%), CD34 (0.16%), CD45 (0.04%), and HLA‐DR (1.64%), and positive for CD73 (91.28%), CD105 (99.97%), and CD90 (100%) (Figure 1F–J), which fulfilled the International Society for Cell Therapy (ISCT) requirements for MSC definition.^52^

### 3.2 Behavioral measurements of rat depression models

**FIGURE 2:**
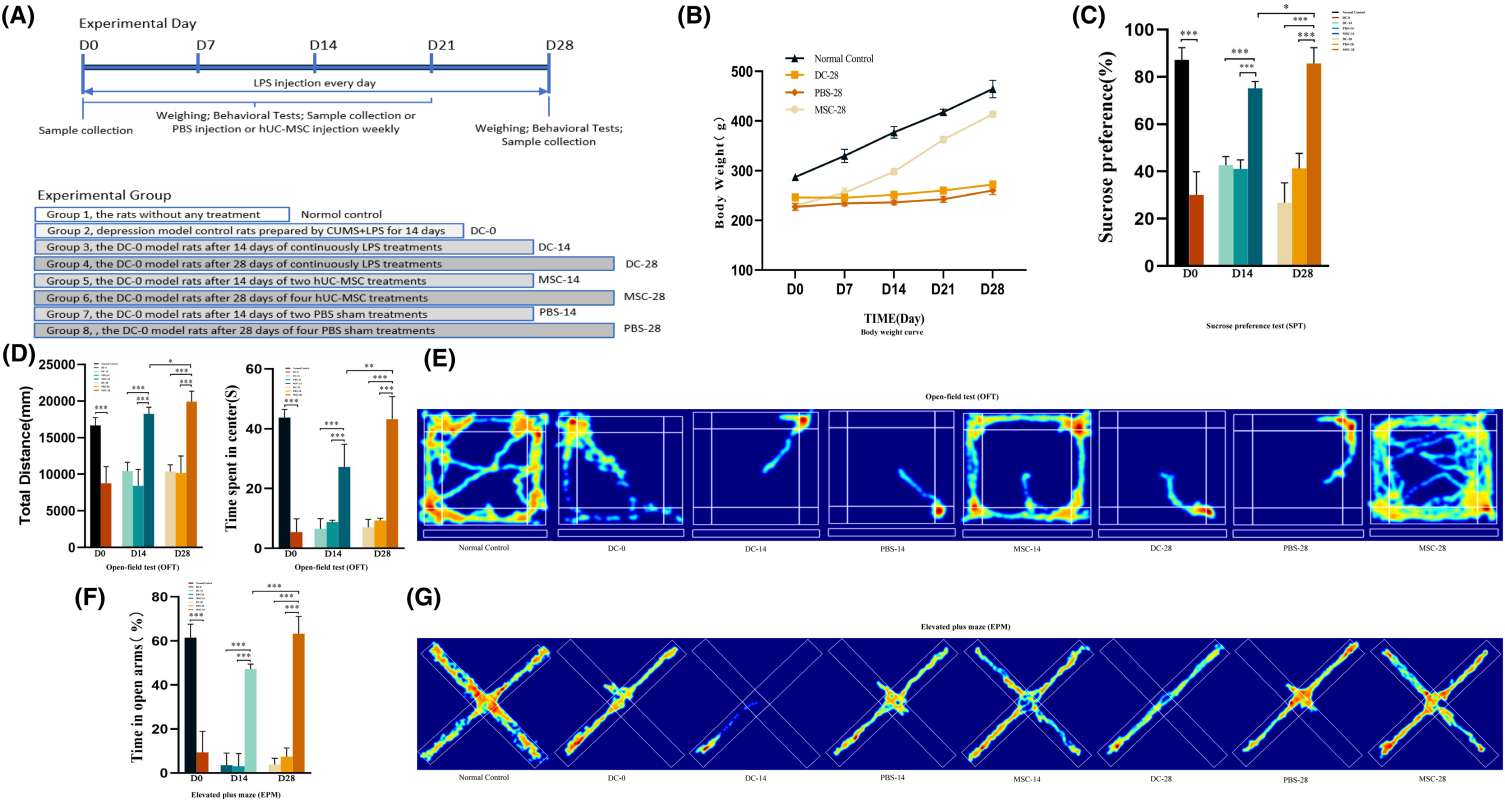
Improvement of rat depressive‐like behaviors by hUC‐MSC treatments. A diagram of the experimental timeline (A). Body weight (BW) of the rats measured weekly in each group (B). Sucrose preference test (SPT) for sucrose consumption from the rats in each group before and after PBS and hUC‐MSC treatments (*n* = 6. *p < 0.05, **p < 0.01, and ***p < 0.001) (C). The total distance and time spent in the center of the OFT (D, E) and EPM (F, G) for the eight groups were tracked before and after PBS and hUC‐MSC treatments with digital cameras and analyzed by the animal behavior analysis system (*n* = 6. *p < 0.05, **p < 0.01, and ***p < 0.001).

The experimental timeline was designed (Figure 2A), and a total of 48 rats (six rats each in eight groups) were randomly assigned to the following eight groups according to the experimental design: normal control, DC‐0, DC‐14, DC‐28, MSC‐14, MSC‐28, PBS‐14, and PBS‐28. Based on the measurements, the body weight (BW) of the rats from DC‐0, DC‐14, and DC‐28 group slowly decreased compared with the normal control group(Figure S2b), and such loss in BW was significantly, although not completely, restored by hUC‐MSC treatments compared with the normal control and PBS groups (*p* < 0.05; Figure 2B). Rat depression models were successfully induced by treatments of CUMS combined with LPS according to the SPT, OFT, and EPM results (Figure 2C–G). The depressed model rats showed significantly decreased sucrose consumption in the SPT (Figure 2C, Figure S2c), and the rats exhibited significant anxiety‐like behaviors compared with the normal controls (*p* < 0.05) including the time spent in the center zone of the OFT (Figure 2D,E, Figure S2d,e) and in the open arms of the EPM (Figure 2F,G, Figure S2f,g). The anxiety‐like behaviors of the depressed rats were significantly reversed by the administration of hUC‐MSCs as predicted. However, this improvement was not observed in the PBS treatment groups (Figure 2D,F). The best result was observed in the MSC‐28 group, wherein the rats received four injections of hUC‐MSCs when compared with two injections in the MSC‐14 group (Figure 2C–G). These results suggested that rat depression behaviors induced by CUMS combined with LPS could be improved by hUC‐MSC treatments. The experiment was repeated 3 times, and 6 rats were used for each group.

### 3.3 Histotomy and immunohistochemistry of hippocampus

**FIGURE 3:**
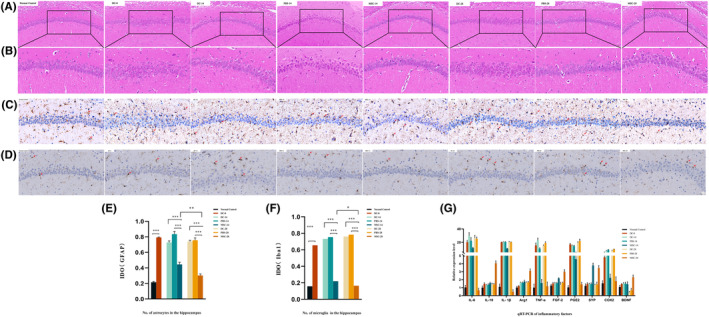
Recovery of hippocampal structure and function in depressed rat models by hUC‐MSC treatments. Morphology of rat hippocampus observed after HE staining, which was derived from NC, DC‐0, DC‐14, PBS‐14, MSC‐14, DC‐28, PBS‐28, and MSC‐28 groups, and the normal and damaged hippocampus was compared among the groups. 100×, scale bar = 100 μm (A1‐8). Image of rat hippocampal CA1 region enlarged from A1‐8 correspondingly, in which the rat hippocampal CA1 region with normal and destroyed neurons was observed. 400×, scale bar = 50 μm (B1‐8). Immunohistochemical images of rat hippocampal CA1 region stained with anti‐GFAP antibody (C1‐8) and anti‐IBA1 antibody (D1‐8), wherein the IBA1‐positive microglia and GFAP‐positive astrocytes were displayed in normal control, DC‐0, DC‐14, PBS‐14, MSC‐14, DC‐28, PBS‐28, and MSC‐28 groups, respectively. 400×, scale bar = 50 μm. The damaged neurons caused by CUMS and LPS treatments in DC‐0 (C2, D2), DC‐14 (C3, D3), and DC‐28 group (C6, D6) were reversed by hUC‐MSC treatments in MSC‐14 (C5, D5) and MSC‐28 (C8, D8) (D). The average IOD of GFAP from immunohistochemical images of normal control, DC‐0, DC‐14, PBS‐14, MSC‐14, DC‐28, PBS‐28, and MSC‐28 groups (E). The average IOD of IAB1 from immunohistochemical images of normal control DC‐0, DC‐14, PBS‐14, MSC‐14, DC‐28, PBS‐28, and MSC‐28 groups (F). Expressions of inflammatory factors in rat hippocampus were quantitated using qRT‐PCR for pro‐inflammatory factors IL‐6, IL‐1β, TNF‐α, PGE2, and COX2 and anti‐inflammatory factors IL‐10, Arg1, FGF2, SYP, and BDNF (*n* = 6 rats per group) (G).

HE staining of rat hippocampal CA1 region derived from the depression rat model before and after hUC‐MSC treatments showed that the morphological structure of the hippocampus was destroyed in the rat depression model DC‐0 (Figure 3A2,B2); DC‐14 (Figure 3A3,B3); and DC‐28 (Figure 3A6,B6) and was improved at 14 and 28 days after first administration of hUC‐MSCs, represented by groups MSC‐14 (Figure 3A5,B5) and MSC‐28 (Figure 3A8,B8), respectively. Better results were obtained from the MSC‐28 group, in which the rats received four injections of hUC‐MSCs. Immunohistochemistry of GFAP, a marker of mature astrocytes, and IBA1, a microglia specific marker, showed that there were more IBA1‐positive and GFAP‐positive cells in rat hippocampal CA1 regions in the DC groups (DC‐0, Figure 3C2,D2; DC‐14, Figure 3C3,D3; DC‐28, Figure 3C6,D6) and PBS groups (PBS‐14, Figure 3C4,D4 and PBS‐28, Figure 3C7,D7) than the normal control (Figure 3C1,D1) and MSC‐treated groups (MSC‐14, Figure 3C5, and MSC‐28, Figure 3C8,D8). Immunohistochemical staining also showed that the hippocampal structure was clearly viewed and with orderly and dense arrangements, and the neurons in rat hippocampal CA1 regions had intact structures with regular morphology in the normal control group. The hippocampal structure was atrophied and loosely arranged, neurons in the rat hippocampal CA1 regions shrank significantly, and the cytoplasm was deeply stained in the DC groups (DC‐0, Figure 3C2,D2; DC‐14, Figure 3C3,D3; DC‐28, Figure 3C6,D6). The staining also showed that some of the neurons were vacuolated, degenerated, and even necrosed. After hUC‐MSC treatment, the number of IBA1‐positive and GFAP‐positive cells in the MSC‐D14 and MSC‐28 groups was obviously decreased, and the neurons in the CA1 regions were rearranged neatly (Figure 3C,D). The average integrated optical density (IOD) of IBA1 and GFAP in the CA1 regions was significantly decreased after hUC‐MSC treatments compared with the DC groups, and more effective results were obtained for the MSC‐28 group rather than the MSC‐14 group (Figure 3E,F). The above results indicated that three doses of hUC‐MSCs can restore hippocampal damage owing to astrocytes and microglial activation caused by CUMS and LPS within 28 days. These data showed that hUC‐MSCs exerted an inhibitory effect on CUMS and LPS‐induced astrocyte and microglial activation.

### 3.4 Quantitative real‐time polymerase chain reaction (qRT‐PCR)

Consistent with morphological changes, qRT‐PCR results showed that expression of pro‐inflammatory factors, IL‐6, IL‐1β, TNF‐α, PGE2, and COX2, in the rat hippocampus was promoted by CUMS and LPS treatments. Conversely, anti‐inflammatory factors, IL‐10, Arg1, FGF2, SYP, and BDNF, were inhibited (Figure 3G, Table 1). Similar to morphological changes, more effective results were seen in the MSC‐28 group at 4 weeks of hUC‐MSC treatment, wherein the rats received four injections of cells (Figure 3G). These results suggested that hUC‐MSC treatments could reverse the inflammatory responses of rat hippocampus induced by CUMS and LPS.

**TABLE 1:** Sequences for primers used in qRT‐PCR.

Gene	Primer sequences
*GAPDH*	Forward: CAACTCCCTCAAGATTGTCAGCAA
	Reverse: GGCATGGACTGTGGTCATGA
*IL‐6*	Forward: CTCTCCGCAAGAGACTTCCA
	Reverse: TCTCCTCTCCGGACTTGTGAA
*IL‐ 1β*	Forward: GTGGCAGCTACCTGTGTCTT
	Reverse: GGAGCCTGTAGTGCAGTTGT
*TNF‐α*	Forward: TGATCGGTCCCAACAAGGA
	Reverse: TGCTTGGTGGTTTGCTACGA
*IL‐10*	Forward: TGCGACGCTGTCATCGATTT
	Reverse: TGACTGAGGTGGTGGCTTTC
*PGE2*	Forward: CAGTCCTGAGACTAATGCGCT
	Reverse: TTACGTTCCTCCAACGAGGC
*COX‐2*	Forward: TGTGAAAGGGTGTCCCTTCG
	Reverse: ACAACACAGGAATCTTCACAAATGG
*BDNF*	Forward: CCGAGCTCATCTTTGCCACA
	Reverse: GAAGCAGCTTTCTCAACGCC
*Arg1*	Forward: GGAAGACAGCAGAGGAGGTG
	Reverse: TATGGTTACCCTCCCGTTGA
*FGF‐2*	Forward: AAGCGGCTCTACTGCAAGAA
	Reverse: GCTGTAGTTTGACGTGTGGG
*SYP*	Forward: CAGTTCCGGGTGGTCAAGG
	Reverse: ACTCTCCGTCTTGTTGGCAC

### 3.5 Transcriptomic profile of rat hippocampus

**FIGURE 4:**
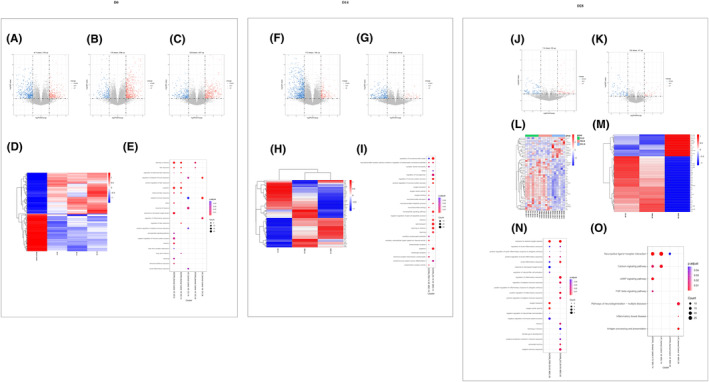
Transcriptomic profile and heatmap of rat hippocampus. The volcano plot for downregulated and upregulated genes derived from DC‐0, DC‐14, and DC‐28 groups that were compared with the normal control group, respectively (A‐C). The heatmap for DEGs derived from DC‐0, DC‐14, DC‐28, and the normal control group was compared (D). The downregulated and upregulated genes are colored in blue and red, respectively. The GO enrichment analysis showed the downregulated and upregulated genes from DC‐0, DC‐14, and DC‐28 groups (E). Volcano plot for downregulated and upregulated genes derived from MSC‐D14 that was compared with the DC‐14 and PBS‐28 groups, respectively (F, G). The heatmap for DEGs derived from the MSC14, PBS‐14, and DC‐14 groups was compared (H). The GO enrichment analysis showed the DEGs after hUC‐MSC treatment for 2 weeks (I). Volcano plot for downregulated and upregulated genes derived from MSC‐28, DC‐28, and PBS‐28 groups that was compared with DC‐28 and PBS‐28 groups, respectively (J, K). The heatmap for DEGs at day 14 of hUC‐MSC treatments for MSC‐14, PBS14, and DC14 and day 28 of hUC‐MSC treatments for MSC‐28, PBS28, and DC‐28 group was revealed, and significant differences between the MSC treatment and PBS or normal control group were observed (L, M). The GO enrichment analysis showed the DEGs after hUC‐MSC treatments for 4 weeks (N). The KEGG engaged in the pathways after hUC‐MSC treatments for 2 and 4 weeks was shown, respectively (O).

The volcano plot of gene expression profiles in rat hippocampi on day 0 (DC‐0, Figure 4A), day 14 (DC‐14, Figure 4B), and day 28 (DC‐28, Figure 4C) after the establishment of the rat depression model showed that 411, 179, and 228 genes were downregulated, and 276, 598, and 407 genes were upregulated, respectively, compared with the normal control. Transcriptomic heatmap of the rat hippocampus indicated that the DC‐0, DC‐14, and DC‐28 groups had similar gene expression pattern but were significantly different from the normal control group (Figure 4D). GO enrichment analysis indicated that the DEGs of DC groups compared to the normal control group were mainly enriched in depression‐related performance, such as learning or memory cognition, fear response, negative regulation of immune system process, acute inflammatory response, oxygen carrier activity, and regulation of oxidoreductase activity (Figure 4E). GO terms with a corrected P.adjust <0.05 were considered significantly enriched among the DEGs. Kyoto Encyclopedia of Genes and Genomes (KEGG) enrichment analysis demonstrated that the DEGs were engaged in neuroactive ligand‐receptor interaction, cAMP signaling pathway, and TGF‐beta signaling pathway, particularly in pathways of neurodegeneration‐multiple disease. When the depressed rats (DC‐14 group) received hUC‐MSC treatments for 2 weeks, 712 genes were downregulated and 185 genes were upregulated compared with the DC‐14 group (Figure 4F), and 276 genes were downregulated and 54 genes were upregulated compared with the PBS‐14 group (Figure 4G). When the depressed rats (DC‐28 group) received hUC‐MSC treatments for 4 weeks, 104 genes were downregulated and 47 genes were upregulated compared with the DC‐28 group (Figure 4J), and 114 genes were downregulated and 83 genes were upregulated compared with the PBS‐28 group (Figure 4K). The heat map showed that there was a significant difference among the MSC‐14, PBS‐14, and DC‐14 groups (Figure 4H; *p* < 0.05). Similar results were observed among the MSC‐28, PBS‐28, and DC‐28 groups (Figure 4I,M). This suggested that hUC‐MSCs could reverse rat gene expressions and treat depression, and the depression was significantly improved compared to the group that received three injections in 28 days based on the gene expression pattern. The GO analysis indicated that DEGs were primarily enriched in learning or memory, positive regulation of acute inflammatory response, negative regulation of immune system process, and reactive oxygen species in the DC, MSC, and normal control groups (Figure 4I,N), while the KEGG analysis revealed that the DEGs were engaged in the pathways related to neuroactive ligand‐receptor interaction, cAMP signaling pathway, and neurodegeneration diseases (Figure 4O). Taken together, RNA sequencing identified that the rat models of depression were successfully created by CUMS and LPS treatment, and such models can be fully recovered with hUC‐MSC treatments in 4 weeks.

### 3.6 Metabolomic profile of rat serum

**FIGURE 5:**
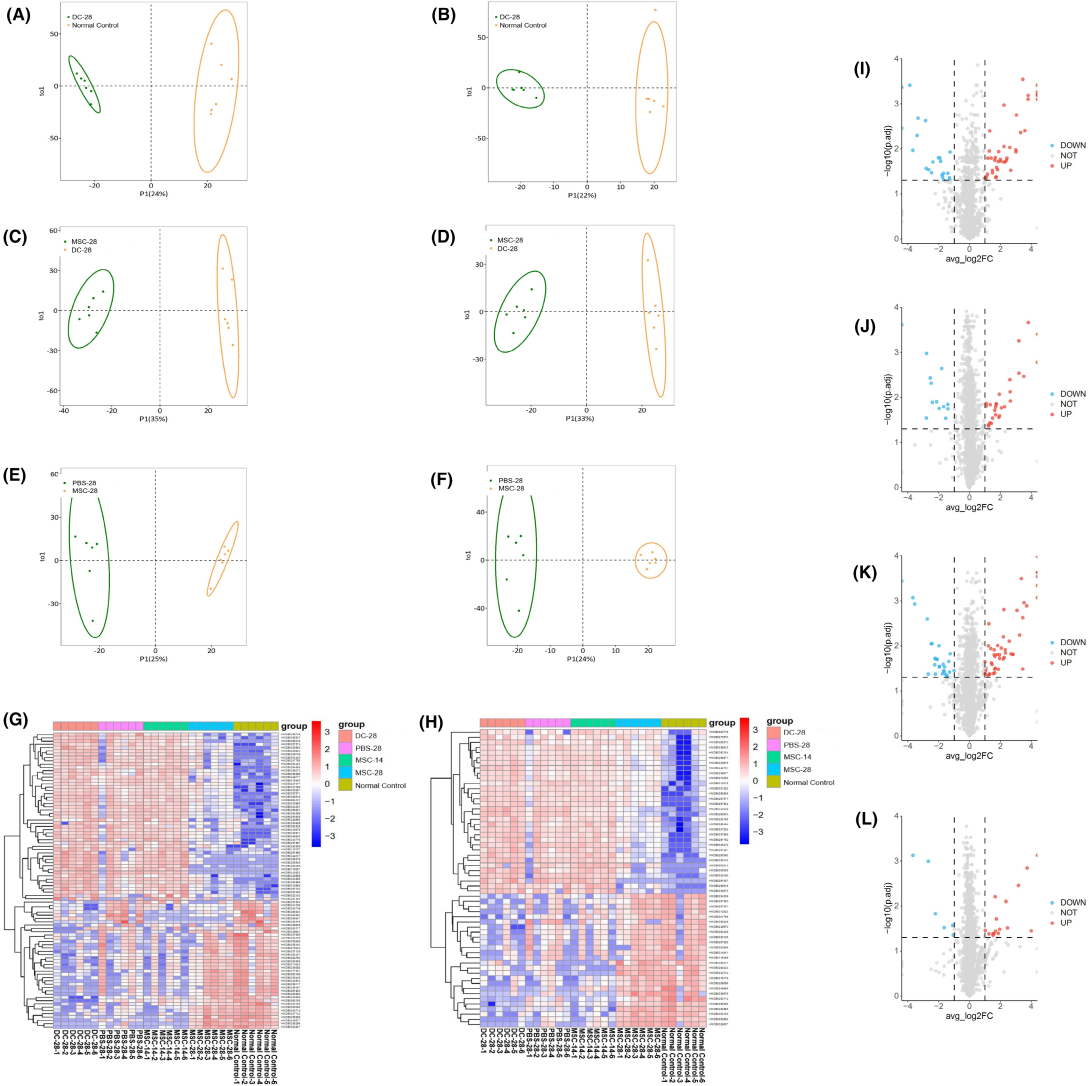
Metabolomic profiles of serum derived from depressed rats before and after hUC‐MSC treatments. OPLS‐DA score plot of normal control and DC‐28 group in the positive (A) and negative ion modes (B). OPLS‐DA score plot of MSC‐28 and DC‐28 in the positive (C) and negative ion modes (D). OPLS‐DA score plot of MSC‐28 and PBS‐28 in the positive (E) and negative ion modes (F). The cluster heatmap of metabolites demonstrated for DC‐28, PBS‐28, MSC‐14, MSC‐28, and normal control groups in positive (G) and negative ion modes (H). The volcano plot showed the up‐ and downregulated metabolites of normal control and DC‐28 groups (I). The volcano plot indicated up‐ and downregulated metabolites of the MSC‐28 and DC‐28 groups (J). The volcano plot indicated up‐ and downregulated metabolites of MSC‐14 and NC (K). The volcano plot indicated up‐ and downregulated metabolites of MSC‐28 and normal control groups (L). The downregulated and upregulated metabolites are colored in blue and red, respectively.

Serum metabolomics in the depressed rat model was compared before and after hUC‐MSC treatments. Serum samples were subjected to analysis using both positive and negative ion electrospray ionization (ESI) modes, respectively. The OPLS‐DA scoring plot from positive and negative ion mode showed that clear segregation and discrimination were observed between the DC‐28 and normal control groups, respectively (Figure 5A,B). Similar results were also observed between the MSC‐28 and DC‐28 and MSC‐28 and PBS‐28 groups (Figure 5C–F). Based on the OPLS‐DA analysis, 94 differential metabolites were identified between the normal control and DC‐28 groups (Figure 5I), 71 differential metabolites were identified between the MSC‐28 and DC‐28 (Figure 5J) groups, and 24 differential metabolites were identified between the MSC‐28 and normal control groups (Figure 5L). Interestingly, 108 differential metabolites were identified between the MSC‐D14 and normal control groups (Figure 5K). All of the above differential metabolites met the criteria of VIP > 1 and *p* < 0.05. These OPLS‐DA score plots indicated that the metabolic profiles between the normal rat and model rat were disturbed by CUMS and LPS stimulation, and the disturbance could be reversed by hUC‐MSC, but not PBS treatment. The cluster heatmaps revealed that metabolites observed in MSC‐D28 were much more similar to the normal control group, but significantly differed from the DC‐28, PBS‐28, and MSC‐14 groups in positive (Figure 5G) and negative ion modes (Figure 5H). The result suggested that depressed rats induced by CUMS and LPS could be reversed with four injections of hUC‐MSCs over 28 days of treatment.

### 3.7 Proteomic profile of rat serum

**FIGURE 6:**
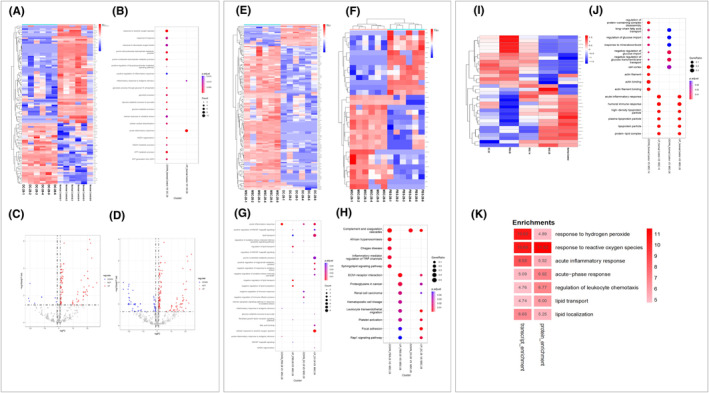
Proteomic profiles of serum derived from depressed rats before and after hUC‐MSC treatments. The differential proteins of serum from normal control, DC‐28, PBS‐28, MSC‐14, and MSC‐28 groups were analyzed by proteomic profiling. Heatmap showed that differential proteins were detected between the DC‐28 and normal control groups (A). The expression levels were indicated by different intensities of colors, which were represented by the color‐key bar with p‐value. Higher level was indicated by red and lower by blue. GO enrichment analysis indicated the DEPs in normal control and DC‐28 groups (B). The volcano plot showed variation of proteins in the normal control and MSC‐14 groups (C). The fold change log (base 2) is on the *X*‐axis, and the negative false log discovery rate (*p*‐value) (base 10) is on the *Y*‐axis. Higher expressions are indicated by red and lower by blue. The volcano plot showed the variation of proteins in the normal control and MSC‐28 groups (D). Heatmap of differential proteins was detected in the DC‐28 and MSC‐28 groups (E). Heatmap of differential proteins was detected in the PBS‐28 and MSC‐28 groups (F). GO enrichment analysis showed that upregulated and downregulated proteins were detected between the DC‐28 and MSC‐28 and the PBS‐28 and MSC‐28 groups, respectively (G). KEGG enrichment analysis showed that differential proteins were mainly enriched in DC‐28, PBS‐28, and MSC‐28 groups (H). The heatmap of differential proteins showed in the normal control, DC‐28, PBS‐28, MSC‐14, and MSC‐28 groups (I). GO enrichment analysis showed that the differential proteins were mainly enriched in the normal control, MSC‐14, and MSC‐28 groups (J). Integration of differential genes and proteins found between depression and normal rat from transcriptomic and proteomic profiles showed that the pathways, hydrogen peroxide, response to reactive oxygen species, acute inflammatory response, lipid transport and lipid localization were engaged in depression (K). Sequences for primers used in qRT‐PCR are listed in Table 1.

The heatmap indicated that there was a significant difference between the DC‐28 and normal control groups (Figure 6A), and 104 differential proteins were identified significantly according to GO enrichment analysis (*p* < 0.05), which were categorized and engaged in the response to reactive oxygen species, glucose catabolic process, acute inflammatory response, and purine ribonucleotide metabolic process (Figure 6B). The volcano plot revealed that 111 and 92 proteins were upregulated, 51 and 8 proteins were downregulated in the MSC‐14 (Figure 6C) and MSC‐28 (Figure 6D) groups compared with the normal control group, respectively. Based on transcriptomic analysis, depressed rats recovered better with hUC‐MSC treatment for 28 days than for 14 days. A similar observation was obtained from the significantly differential proteins between the MSC‐28 and DC‐28 groups (Figure 6E) and between the MSC‐28 and PBS‐28 groups (Figure 6F). The differential proteins were significantly enriched in complement and coagulation cascades, inflammatory mediator regulation of transient receptor potential (TRP) channels, and leukocyte transendothelial migration (TEM; Figure 6G,H). The heatmap clearly showed that the differential proteins in MSC‐28 were much closer to the normal control group (Figure 6I). GO enrichment showed that the differential proteins caused by hUC‐MSC treatment in the MSC‐14 and MSC‐28 groups were enriched in identical pathways (Figure 6J). Enrichment analysis of the differences between the MSC‐28 and DC‐28 groups showed that differential genes and proteins mainly involved in seven pathways, hydrogen peroxide, response to reactive oxygen species, acute inflammatory response, lipid transport, and lipid localization, were depression‐related (Figure 6K). The result indicated that rat depression could be reversed by hUC‐MSC treatments.

## 4 DISCUSSION

Depressive disorder, also known as depression, is a common psychological disorder and can cause difficulties in all aspects of life. Depression results from a complex interaction of social, psychological, and biological factors.^53^ It is well known that people who have lived through abuse, severe losses, or other stressful events are more likely to develop depression than others.^54^ However, the pathogenesis of depression caused by stress is poorly understood. Although genome‐wide association analyses of huge population identified 44 risk variants involved in major depression, genetics alone do not account for most of the risk of major depression. Extensive evidence now suggests that psychological stress can trigger significant increases in inflammatory activity. This inflammation can in turn elicit the changes of depressive behavior, such as sadness, anhedonia, fatigue, psychomotor retardation, and social‐behavioral withdrawal.^55^ It is now an accepted fact that stressful conditions can induce psychiatric disorders mediated by immune system response. Animal models of affective illness may not fully match the human condition, and numerous animal models have been used to screen antidepressant drugs.^56^ CUMS appears to be more reliable and effective to establish depression models than surgery, acute stress, tail suspension, and forced swimming.^57^ CUMS is currently the most commonly used, reliable, and effective rodent model of depression.^58^ However, the rat depression model established by CUMS stimulations in this study showed behavioral instability and recovered spontaneously after cessation of stimulation even if some died after such treatments (unpublished data). Animal models of depression‐like behavior also have been induced by LPS administration, more than 20 years ago.^59^ Because LPS administration likely activates various pathways associated with depression and induces neuronal alterations in brain areas related to mood regulation,^60^ LPS‐induced model has been increasingly used to understand the neurobiology and treatment of depression. Nevertheless, single‐dose LPS‐induced models cannot always separate LPS‐induced depression‐like behavior from sickness behavior. Repeated and intermittent administration of LPS to induce the basic model of depression requires 4 months. Hence, we attempted to use LPS combined with CUMS to induce rat depression models in this study. Rat depression models were successfully established by gentle CUMS treatments combined with repeated low dosages of LPS administration. The resultant rats exhibited pleasure loss, anxiety, tension, and behavioral despair, and such models represented reliable and long‐lasting depressive‐like behaviors.

Microglia, the principal immune cells in the brain, play crucial roles in immune surveillance and maintenance of brain homeostasis. Under stress or pathological conditions, microglia are activated to secrete inflammatory factors that destroy neuronal function, impair neurogenesis, increase susceptibility to stress, and lead to the occurrence and development of depression.^61^, ^62^ Thus, we explored the subsets of astrocytes and microglia in the hippocampus. We explored the changes in pattern, number, and structure of astrocytes and microglia in the hippocampus through immunohistochemistry. In accordance with the expected results, microglia and astrocytes were activated by CUMS and LPS treatment that resulted in cell vacuolation, degeneration, necrosis, and even an increase in the number of cells. Correspondingly, the hippocampus showed significant atrophy, loose arrangement, and shrinkage in CA1 regions compared with normal rats. Accompanied with activation of microglia and astrocytes, the expression of pro‐inflammatory factors such as interleukin (IL)‐1ß, IL‐6, tumor necrosis factor (TNF)‐α, prostaglandin E2 (PGE2), and cyclooxygenase 2 (COX2) was increased. These results indicated that neuronal damage and expression of pro‐inflammatory factors triggered by CUMS and LPS treatments could induce depression‐like behaviors in rats. More importantly, such depression has been reversed with hUC‐MSC treatments particularly in 4 weeks. More uniform arrangement was observed, and the fragmented and dissolved nuclei had significantly improved in hippocampal tissue. The results verified that hUC‐MSCs can modulate inflammatory response and have clinical potential to treat depression.^63^, ^64^ Our findings suggest that hUC‐MSCs have the remarkable ability to reduce inflammation and modulate cytokine production, which is responsible for their therapeutic effects. Additionally, to our knowledge, this was the first time that fluorescent‐labeled hUC‐MSCs had been observed in the hippocampal tissue of depressed rats at 7 days of intravenous injection, except for in the lung, liver, and spleen (Figure S3). Interestingly, the labeled hUC‐MSCs were only detected in damaged hippocampal tissue but not in normal brain tissue (Figure S4). These results further indicated that the hUC‐MSCs had migrated into the site of inflammation in the brain through the BBB, which might be damaged by microglial activation induced by depression. The detailed mechanisms of hUC‐MSCs to repair damaged tissues and resist LPS damage remain to be investigated.

Indeed, stress induces depressive behaviors by triggering inflammation of the brain and peripheral immune system.^65^ Inflammatory changes in depression are characterized by increased expression of pro‐inflammatory factors and decreased expression of anti‐inflammatory factors. For example, the expression of pro‐inflammatory cytokines such as IL‐6 and TNF‐α was increased, and the expression of anti‐inflammatory cytokines such as IL‐10 and TNF‐β was decreased.^65^, ^66^ Recently, more cytokines, their receptors, and ligands associated with depression behaviors have been studied. Among these factors, this study also confirmed that the expression of pro‐inflammatory cytokines—IL‐1ß, IL‐6, TNF‐α, PGE2, and COX2^67^, ^68^—was dramatically increased, but not those of the anti‐inflammatory cytokines, IL‐10, arginase‐1 (ARG1), fibroblast growth factor (FGF)‐2, synaptophysin (SYP), and brain‐derived neurotrophic factor (BDNF) in the rat depression models. As expected, the expressions of pro‐inflammatory cytokines were significantly reduced and the anti‐inflammatory cytokines were dramatically increased after hUC‐MSC treatments. Because the depression model rats used in this study continually received LPS administration, the model rats could reliably maintain the depression behaviors during hUC‐MSC treatment. These results indicated that not only the depression behaviors of the modeled rats could be reversed by hUC‐MSC treatments confirmed by others^69^, ^70^ but also the expression of anti‐inflammatory cytokines was triggered, and such changes could fully counteract the damage to the rat hippocampus caused by LPS stimulation. According to the cytokine hypothesis, the cytokine balance is initially destroyed by stress and sympathetic nervous system activation. In turn, increased expression of cytokines is engaged in the neurotransmitter depletion pathway, neuroendocrine pathway, and neural plasticity pathway. Finally, multiple interactions between these pathways result in depression behaviors. Our results support the cytokine hypothesis of depression, although the hypothesis is still controversial. This is consistent with the theory that the mechanism of depression is related to inflammation.^71^

Transcriptomics, proteomics, and metabolomics have been commonly applied in the investigation of complex and concomitant diseases. Few attempts have been reported on depression disorders. Here, transcriptomics, proteomics, and metabolomics were performed to verify the therapeutic effects of hUC‐MSCs on depression model rats. Transcriptomic analysis was consistent with the results of qRT‐PCR for inflammatory factors in this study, which further confirmed that the rat model of depression was successfully established by a combination of CUMS and LPS treatment for 14 days. Compared with the normal control group, 411, 179, and 228 downregulated genes and 276, 598, and 407 upregulated genes were detected in the hippocampus of rats subjected to CUMS combined with LPS treatment for 14 days (DC‐0), followed by continuous injections of LPS for 14 days (DC‐14) and continuous injections of LPS for 28 days (DC‐28), respectively. More DEGs were detected in this study than in another report that used CUMS in the same rat strain. It is thought that additional LPS administration can trigger more gene expression. Endotoxin tolerance, a protective response against gram‐negative bacterial infection, is well verified, and LPS tolerance also occurred in this study even when the tolerance was partially suppressed after continuous LPS injection. Endotoxin tolerance, regarded as the regulatory mechanism of the host against inflammation, is a complex pathophysiological process and involved in multiple signaling pathways.^72^ The main characteristics of endotoxin tolerance are downregulation of inflammatory mediators such as TNF‐α and IL‐1β, and C‐X‐C motif chemokine 10 (CXCL10) and upregulation of anti‐inflammatory cytokines such as IL‐10 and TGF‐β. Similarly, only the anti‐inflammatory cytokines IL‐10, ARG, FGF‐2, SYP, and BDNF were upregulated at different levels after repeated LPS administration, but not pro‐inflammatory factors, IL‐1ß, IL‐6, TNF‐α, PGE2, and COX2 (Figure 3F). We propose that sustained neuronal damage caused by continuous injections of LPS prevents the neuroimmune system from fully reversing the inflammatory response. The molecular mechanisms of LPS tolerance regulated from pro‐inflammatory to anti‐inflammatory state still need more data for validation. The GO enrichment analysis showed that the DEGs between depressed and normal rats were mainly enriched in the categories of depression related to learning or memory, cognition, fear response, neurotransmitter secretion, hypoxia response, and inflammatory response regulation. KEGG analysis revealed the DEGs were focused on neuroactive ligand‐receptor interaction, calcium signaling pathway, cAMP signaling pathway, TGF‐beta signaling pathway, neurodegeneration multiple diseases, inflammatory bowel disease, and antigen processing and presentation, which are related to depression (Figure 4J). Here, the therapeutic potential of hUC‐MSCs has been highlighted by transcriptomic analysis of the depressed rat hippocampus before and after hUC‐MSC treatments. Our results indicated that the depression behaviors and transcriptomics of the model were reversed to normal or close to normal (Figure 4M,N).

Although the depression was improved based on the expression levels of inflammatory factors and transcriptome analysis of rats treated with two or four injections of hUC‐MSCs, satisfactory results were obtained from the group received four injections of hUC‐MSC in 4 weeks. Based on the above findings, proteomic and metabolomic analysis for differential proteins and metabolites have been focused on normal control, depression, PBS‐, and hUC‐MSC‐treated rats at 4 weeks after treatment. GO analysis identified that the hUC‐MSC treatment engaged in reactive oxygen species, acute inflammatory response, memory, and leukocyte‐mediated immunity. These findings are consistent with the underlying pathogenesis of depression, which further confirmed that hUC‐MSCs have the potential to improve depressive symptoms in rats. The proteomics and metabolomics data derived from rat serum in this study were similar to those obtained from the hippocampal tissue.^73^, ^74^ Integrated analysis of proteomics and metabolomics indicated that, similar to other reports but different, significant differences were enriched in response to hydrogen peroxide, reactive oxygen species, acute inflammatory response, acute‐phase response, regulation of leukocyte chemotaxis, lipid transport, and lipid localization.^75^ This might be because the rat serum was employed for proteomics and metabolomics. Nevertheless, the proteomic and metabolomic results also suggested that hUC‐MSCs are an alternative therapeutic option for depression. The present study verified that hUC‐MSC infusion can restore depression induced by CUMS combined with LPS in rats. Through a systematic literature search, this result has been confirmed not only by histological and immunohistochemical analysis but also for the first time by comparation of transcriptomic, proteomic, and metabolomic changes in this study. The detailed pathological mechanisms of transcription factors, protein molecules, and metabolites involved in depression need to be further investigated.

## 5 CONCLUSION

Rat depression models could be well created by treatments of CUMS combined with LPS. Such models had stable and reliable depression behaviors and transcriptomic profiles under continuous LPS administration compared with normal controls. The behaviors and transcriptomic profiles of the depression‐modeled rats could be reversed by infusion of hUC‐MSCs through the caudal vein. The therapeutic potential of hUC‐MSCs to rat depression had also been verified by proteomic and metabolomic analysis of rat sera before and after treatments. Additionally, it was the first report wherein fluorescent‐labeled hUC‐MSCs had been observed in the hippocampus of depressed rats after intravenous injection except for in the lung, liver, and spleen. Interestingly, the labeled hUC‐MSCs were only detected in damaged hippocampal tissue but not in other brain tissues of depressed rats. We proposed that hUC‐MSCs were an alternative option for depression treatment.

## 6 SHORTCOMINGS AND LIMITATIONS

Data from our present study have provided compelling evidence that hUC‐MSCs exerted anti‐depressive effects and alleviated the behavioral symptoms of depression in rats by inhibiting stress‐induced activation of the immune response and inflammatory response. However, the DEGs, proteins, and metabolites have not been fully verified. The molecular mechanism of hUC‐MSC therapeutic potential remains to be further investigated. The precise mechanisms of antidepressant‐like effects of hUC‐MSCs also remain to be elucidated.

## AUTHOR CONTRIBUTIONS

Siqin Bao, Jitong Guo, and Xihe Li had full access to all the data in the study and take responsibility for the integrity of the data and the accuracy of the data analysis. Jitong Guo conceived and designed the study. Yongli Song undertook the statistical analyses. Pengxiang Wang wrote the first draft of the manuscript. Yunxia Li is the study guarantor. Yuan Gao, Chunxia Hao, and Yang Zhou interpreted data, reviewed the paper, and made critical revision of the manuscript for important intellectual content. All authors read and approved the final version of the manuscript.

## FUNDING INFORMATION

This study was funded by National Natural Science Foundation of China (No. 32060176), National Key Research and Development Program of China (No. 2022YFD1302202), Inner Mongolia Natural Science Foundation of China (No. 2021LHMS08056), and Hohhot Science & Technology Plan (No. 2020‐Ke Ji Xing Meng‐Independent innovation Centre‐06).

## CONFLICT OF INTEREST STATEMENT

The authors declare that they have no financial interests or other conflicts of interest.

## Supporting information


Figure S1.

Figure S2.

Figure S3.

Figure S4.

## Data Availability

The data that support the findings of this study are available from the corresponding author upon request.
