# Supplementary figures and images for: Human umbilical cord mesenchymal stem cells reverse depression in rats induced by chronic unpredictable mild stress combined with lipopolysaccharide
Source: CNS Neurosci Ther. 2024 Mar 3;30(3):e14644. doi: 10.1111/cns.14644 (PMC10909725; doi:10.1111/cns.14644)

Full unedited gel/blot for Figure S1b

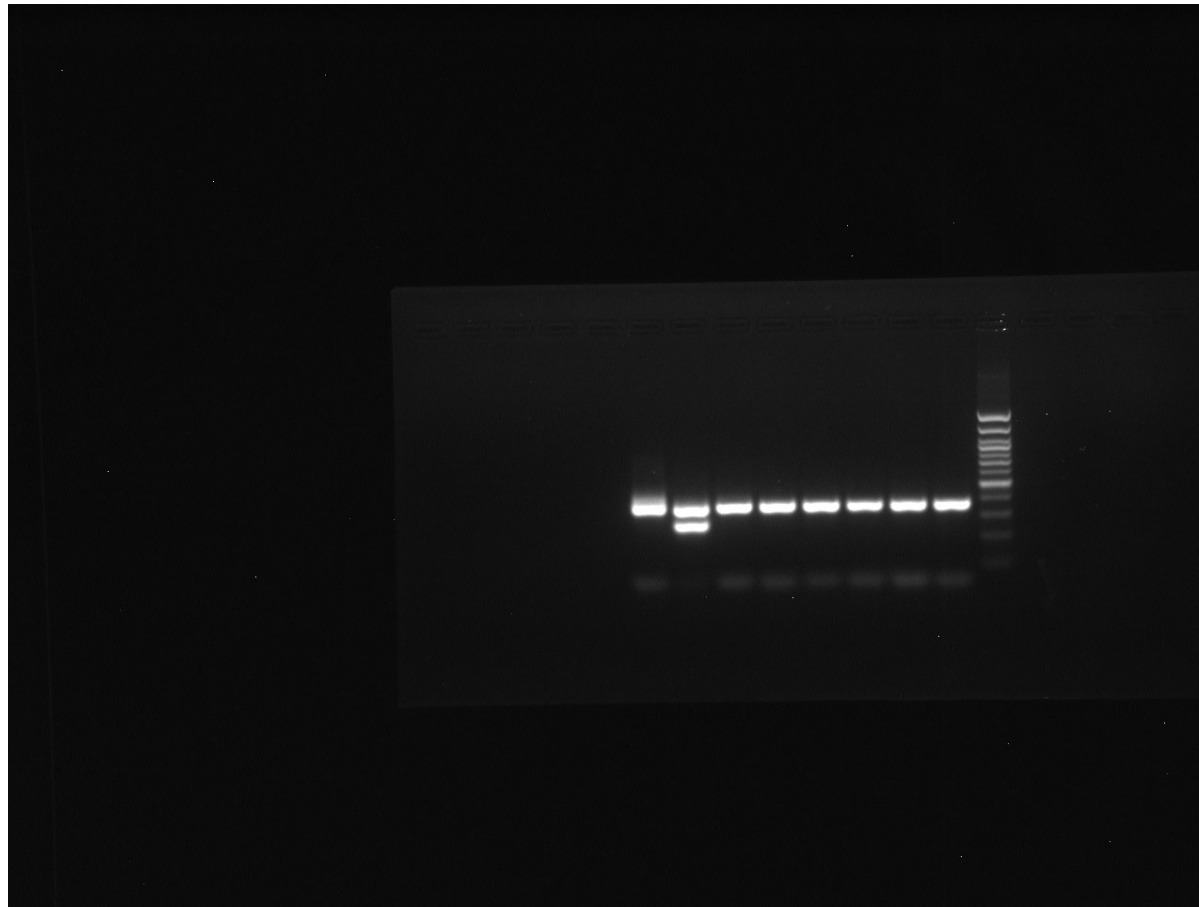

Supplement: Supplementary file 1 — Figure S1. [file CNS-30-e14644-s001.zip › Full unedited gel for Figure S1b_3.pdf]

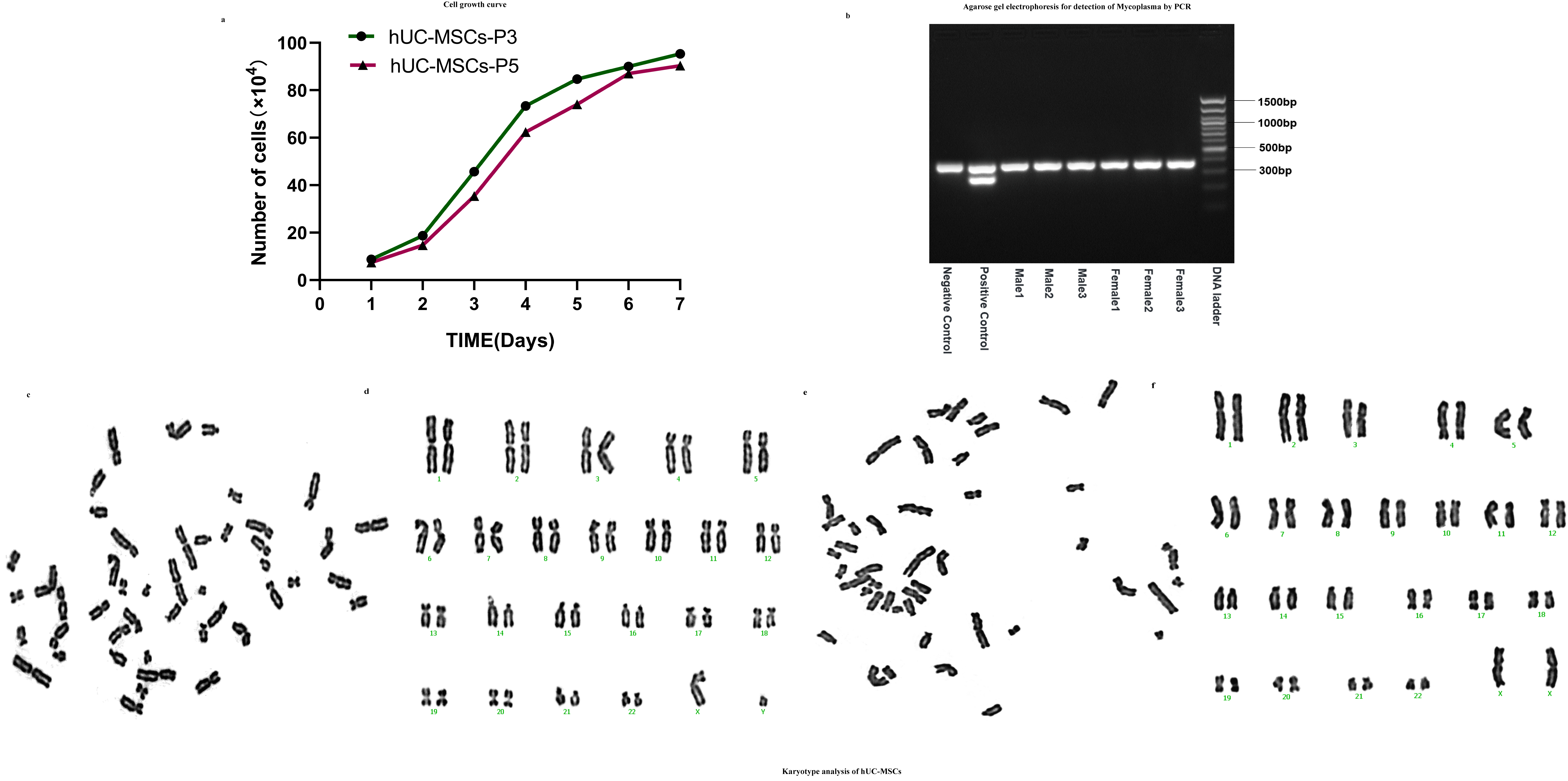

Supplement: Supplementary file 1 — Figure S1. [file CNS-30-e14644-s001.zip › Supplement Fig.1.tif]

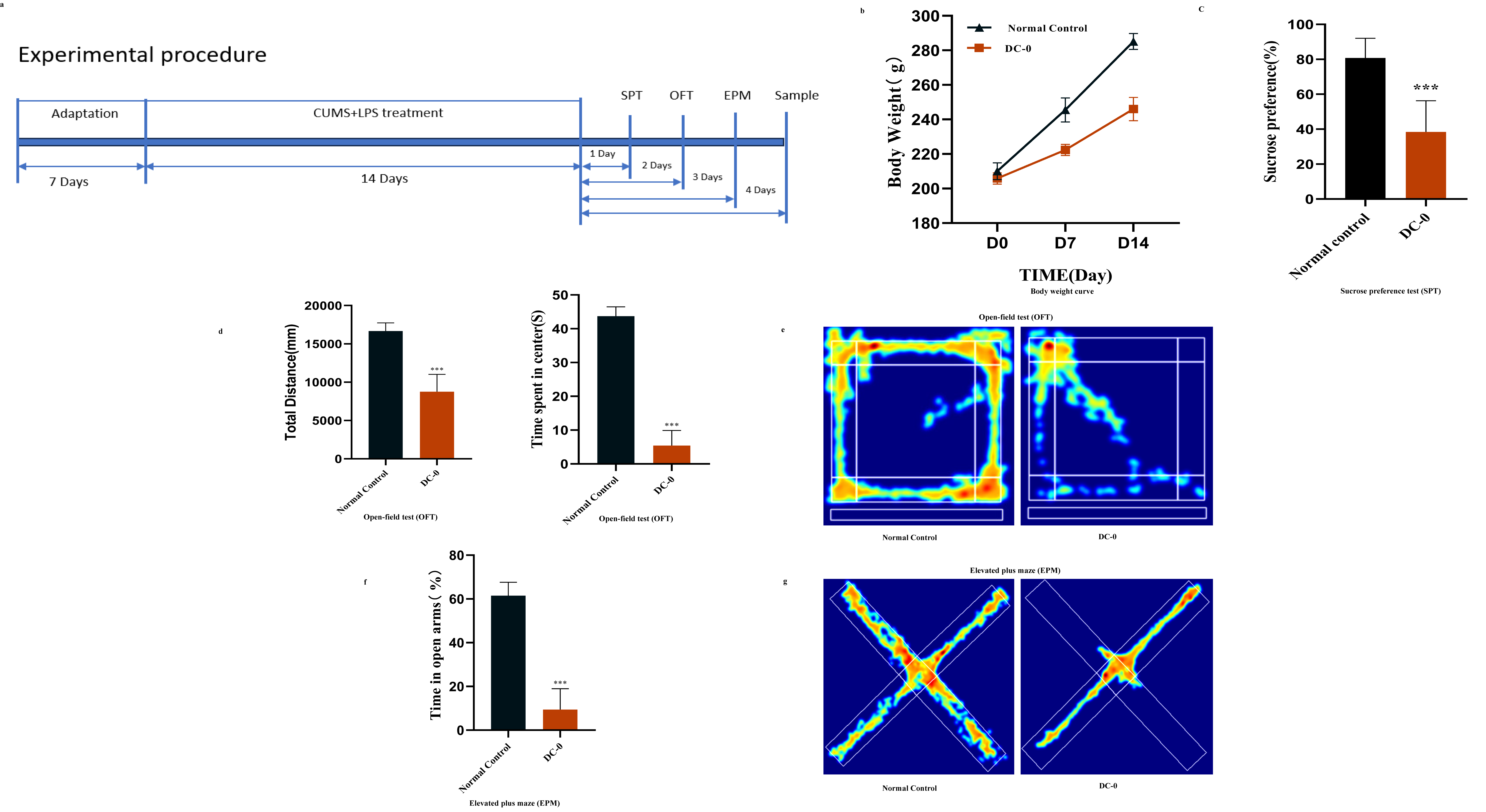

Supplement: Supplementary file 2 — Figure S2. [file CNS-30-e14644-s004.tif]

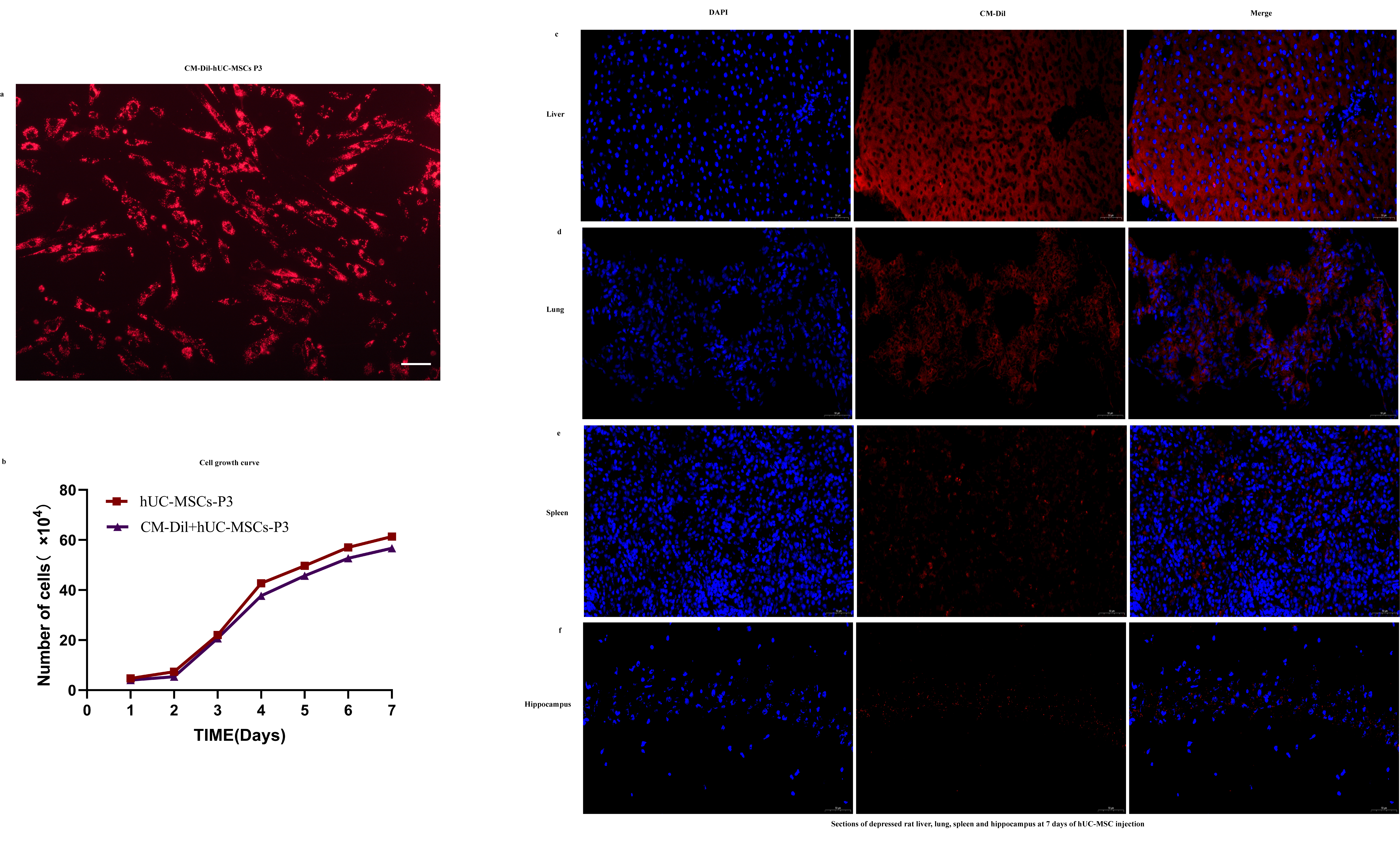

Supplement: Supplementary file 3 — Figure S3. [file CNS-30-e14644-s002.tif]
